# Role of Benzoic Acid and Lettucenin A in the Defense Response of Lettuce against Soil-Borne Pathogens

**DOI:** 10.3390/plants10112336

**Published:** 2021-10-29

**Authors:** Saskia Windisch, Anja Walter, Narges Moradtalab, Frank Walker, Birgit Höglinger, Abbas El-Hasan, Uwe Ludewig, Günter Neumann, Rita Grosch

**Affiliations:** 1 Department of Nutritional Crop Physiology, Institute of Crop Sciences, University of Hohenheim, 70599 Stuttgart, Germany; Anja.Walter2@gmx.de (A.W.); n.moradtalab@uni-hohenheim.de (N.M.); u.ludewig@uni-hohenheim.de (U.L.); guenter.neumann@uni-hohenheim.de (G.N.); 2 Central Chemical-Analytical Laboratory, Institute of Phytomedicine, University of Hohenheim, 70599 Stuttgart, Germany; frank.walker@uni-hohenheim.de (F.W.); birgit.hoeglinger@uni-hohenheim.de (B.H.); 3 Department of Phytopathology, Institute of Phytomedicine, University of Hohenheim, 70599 Stuttgart, Germany; aelhasan@uni-hohenheim.de; 4 Programme Area Plant-Microbe Systems, Leibniz Institute of Vegetable and Ornamental Crops (IGZ) e.V., 14979 Großbeeren, Germany; grosch@igzev.de

**Keywords:** lettuce, root exudates, plant health, phytoalexin, lettucenin, benzoic acid, defense reaction

## Abstract

Soil-borne pathogens can severely limit plant productivity. Induced defense responses are plant strategies to counteract pathogen-related damage and yield loss. In this study, we hypothesized that benzoic acid and lettucenin A are involved as defense compounds against *Rhizoctonia solani* and *Olpidium virulentus* in lettuce. To address this hypothesis, we conducted growth chamber experiments using hydroponics, peat culture substrate and soil culture in pots and minirhizotrons. Benzoic acid was identified as root exudate released from lettuce plants upon pathogen infection, with pre-accumulation of benzoic acid esters in the root tissue. The amounts were sufficient to inhibit hyphal growth of *R. solani* in vitro (30%), to mitigate growth retardation (51%) and damage of fine roots (130%) in lettuce plants caused by *R. solani*, but were not able to overcome plant growth suppression induced by *Olpidium* infection. Additionally, lettucenin A was identified as major phytoalexin, with local accumulation in affected plant tissues upon infection with pathogens or chemical elicitation (CuSO_4_) and detected in trace amounts in root exudates. The results suggest a two-stage defense mechanism with pathogen-induced benzoic acid exudation initially located in the rhizosphere followed by accumulation of lettucenin A locally restricted to affected root and leaf tissues.

## 1. Introduction

Crops are exposed to a great variety of soil microorganisms, which can act and interact as competitors, predators and pathogens [1], but also support nutrient acquisition and stress resilience of the host plant. Losses of yield and product quality, caused by soil-borne pathogens, are among the most limiting factors in plant production [2]. However, efficient control strategies, e.g., against widespread fungal soil-borne pathogens, such as *Rhizoctonia* spp., *Fusarium* spp., *Pythium* spp. or *Olpidium* ssp., are limited due to the long-term persistence of these pathogens in the soils [3,4,5] and poorly available resistant cultivars [6]. Adverse eco-toxicological effects of chemical fungicides urge the establishment of alternatives for disease management [7]. The development of environmentally friendly agricultural management strategies, promoted by beneficial plant–microbe interactions in the rhizosphere is a major focus of recent research activities [8]. In this context, adaptations of plants developing physical and chemical barriers against pathogen attack are of particular interest [9]. Root exudates and plant metabolites with antimicrobial, insecticidal or allelopathic properties are described as an important component of plant defense against pathogens, pests and competitors [10,11,12]. Therefore, the role of root exudates and their interactions with soil microbiota for performance, stress resilience and productivity of plants is intensively studied [10,13,14,15]. However, characterization and quantitative determination of root exudates and their functions under real rhizosphere conditions in soil-grown plants is technically difficult, if not impossible [16]. In this regard, there are numerous interfering factors, such as rapid microbial degradation of root exudates, spatial and temporal variation in release patterns, chemical reaction with other organic compounds in the soil and adsorption to the soil matrix [16]. Therefore, concepts investigating the role of root exudates in rhizosphere interactions are frequently based on correlative observations originating from experiments conducted under controlled conditions, i.e., hydroponic culture, extraction of rhizosphere soil, analyses of soil solutions from microlysimeter studies in minirhizotrons and examination of microbial dynamics using molecular tools [16,17,18,19]. 

Recent studies on a model pathosystem with lettuce (*Lactuca sativa*) and *Rhizoctonia solani* have shown a positive correlation of the antimicrobial compound benzoic acid, detected in the rhizosphere soil solutions in minirhizotron experiments with the soil suppressive potential against *R. solani* [20]. A similar positive correlation between benzoic acid content in the rhizosphere and the biocontrol activities of selected bacterial inoculants (*Pseudomonas* sp. RU47 and *Serratia plymuthica 3Re-4-18*) against the fungal pathogen were also detectable in model experiments and under field conditions [17,20,21]. Accordingly, the role of root-secreted benzoic acid in plant defense modified by the presence of beneficial rhizosphere microorganisms in the lettuce rhizosphere has been discussed [20,22].

Benzoic acid is one of the oldest chemical preservatives, especially used in the cosmetic, drug and food industries [23,24]. Furthermore, this compound has been described as an antimicrobial and (auto)-allelopathic compound in root exudates of various plant species, such as barley, peanut, strawberry, tobacco and also lettuce [18,25,26]. Additionally, benzoic acid can be released by various bacteria and fungi [26,27,28]. Aromatic carboxylates such as benzoic acid have been shown to inhibit or kill microorganisms (i) by interfering with the permeability of the microbial cell membrane, causing uncoupling of both substrate transport and oxidative phosphorylation, (ii) by disruption of intracellular pH homeostasis and (iii) specific inhibition of various enzyme activities [23]. Moreover, defense priming with protective effects against early blight (*Alternaria solani*) in tomato has also been reported for exogenous application of benzoic acid [29].

However, investigations on root exudation of benzoic acid in lettuce have never been conducted under axenic conditions [17,18,20,22]. Therefore, it is still poorly understood whether this compound is released from plant roots or accumulates in the rhizosphere solution as rhizosphere product of microbial origin. In this study, we addressed the hypothesis that benzoic acid is released from lettuce roots in response to pathogen infection by *R. solani* and accumulates in the rhizosphere in pathogen suppressive concentrations. 

Apart from benzoic acid, sesquiterpene phytoalexins (lettucenins) have also been described as antimicrobial secondary metabolites in lettuce, accumulating in the leaf tissue upon exposure to microbial pathogens (i.e., *Fusarium oxysporum*, *Pythium aphanidermatum* and others) but also as a response to membrane damage after chemical elicitation with CuSO_4_ or AgNO_3_ or abiotic stress [30,31,32,33]. Lettucenins and related sequiterpene lactones have been described as a major group of secondary metabolites in lettuce and the whole family of the Asteraceae. They possess multiple functions as antimicrobial compounds acting via cell wall and membrane disruption, as allelopathics, antifeedants and exhibit beneficial medical properties (reviewed by Chadwick et al. [34]). Therefore, we addressed the hypothesis that lettucenins, so far only detected in leaves, could also play a role in pathogen defense responses in lettuce rhizosphere. 

## 2. Results

### 2.1. Root Exudation of Benzoic Acid

To address the question whether benzoic acid, detected in rhizosphere soil solutions of soil-grown lettuce plants in previous studies [17,20,22,25] was released from plant roots or alternatively produced by rhizosphere microorganisms, lettuce was cultivated in a soil-free hydroponic culture system. Intensive microbial root colonization was reduced by frequent replacement (2-day intervals) of the nutrient solution prepared with demineralized membrane-filtered water, since a completely axenic culture of plants in hydroponics is technically difficult and not reliable for longer time periods required for the experiment.

**Figure 1:**
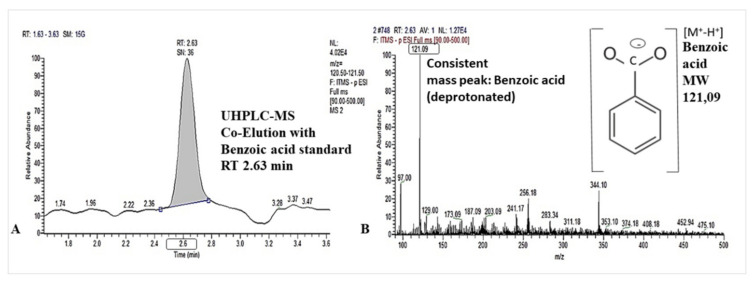
UHPLC-MS separation of benzoic acid in root washings of lettuce at 2.63 min retention time (**A**) and mass spectrum with [M+-H+], base peak at m/z= 121.09 (**B**).

A successful detection of benzoic acid in root washings of hydroponically grown lettuce by UHPLC-MS (Figure 1; Table 1) was indicative of root exudation. 

Further analyses detected benzoic acid also in the root tissue of the lettuce plants used for exudate collection. Free benzoic acid was detectable only in trace concentrations in methanolic root extracts, but significant amounts were recorded after alkaline hydrolysis of the extracts (Table 1), demonstrating that benzoic acid was present in the root tissue in conjugated form as benzoic acid esters. 

### 2.2. Antimicrobial Activity of Benzoic Acid under Realistic Rhizosphere Concentrations

**Figure 2:**
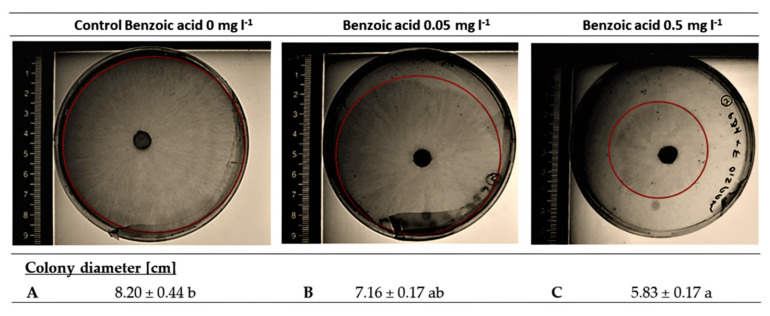
Mycelial growth of *R. solani* AG1-1B after 72 hours on PDA medium, spiked with 0.0 mg L^−1^ (**A**), 0.05 mg L^−1^ (**B**) and 0.5 mg L^−1^ (**C**) of benzoic acid. The red outline indicates the diameter of the mycelial growth. Means ± SE of three replicates per treatment. Different lowercase letters indicate significant differences between treatments by one-way ANOVA, Tukey’s test (*p* ≤ 0.05).

Earlier studies demonstrated a relationship between benzoic acid exudation and disease severity caused by *R. solani* [20]. However, so far, a causal relationship has not yet been demonstrated since it is still unknown whether the rhizosphere concentrations measured for benzoic acid in lettuce plants are sufficient to exert any inhibitory effects on growth and pathogenesis of *R. solani*. Based on the benzoic acid concentration determined in the rhizosphere of lettuce grown on a loamy sand with a suppressive potential against *R. solani* [22], a benzoic acid concentration of approx. 500 µg L^−1^ was calculated for the rhizosphere soil solution at a distance of 1 mm from the root surface. The addition of benzoic acid with the respective concentration in a PDA plating assay with *R. solani* resulted in a significant reduction (30%) of mycelial growth during 72 h. Even 0.05 mg L^−1^ still mediated a reduction of 12%, demonstrating the inhibitory potential of benzoic acid in a concentration range detectable in the rhizosphere of soil-grown lettuce plants (Figure 2).

### 2.3. Protective Role of Benzoic Acid in a Lettuce-R. solani Pathosystem

**Figure 3:**
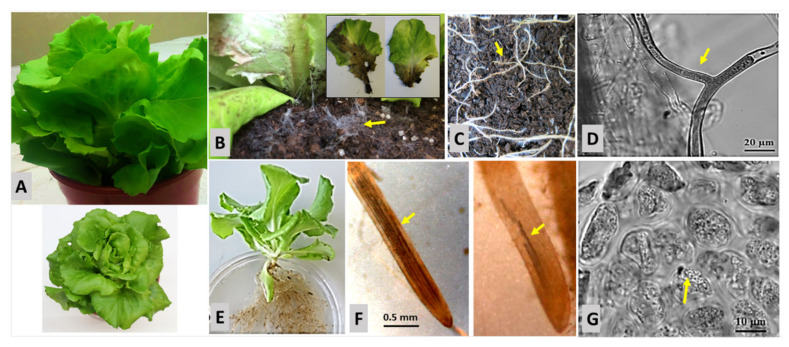
(**A**) Control plant of lettuce, non-infected by *R. solani* and *Olpidium* sp. (**B**) Stem base with browning of lower leaves (**C**) and stunted top-soil fine roots of lettuce (cv. Tizian) infected by *R. solani* AG1-IB (**D**) with fungal hyphae showing rectangular branching (**E**). Root system (**F**) and fine root tips of lettuce seedlings infected by naturally occurring *Olpidium* sp. with typical dark colored lines in xylem. (**G**) Sporangia formation in the root tissue by *Olpidium* sp.

Under optimum conditions for *R. solani* infection, using a peat culture substrate–sand mixture with high porosity to support hyphal spreading and an incubation temperature of 25 °C, first mycelium growth and characteristic bottom rot symptoms on stems and lower leaves were visible in lettuce plants already two days after inoculation (Figure 3B). Additionally, fine roots were preferentially affected in the topsoil by stunted growth and browning (Figure 3C). *Rhizoctonia*-typical rectangular hyphal branching was observed on infected roots (Figure 3D).

Ten days after *R. solani* inoculation, the total biomass of infected plants was significantly reduced by 44.2 g, as compared with the untreated control with a biomass of 164.8 g. Benzoic acid was applied in real rhizosphere concentrations (as determined by Windisch et al. [22]). Application of benzoic acid in three doses with the irrigation water (each 110 µg kg^−1^ substrate throughout the ten days culture period), resulted in a significant suppressive effect (51%) on *R. solani*-induced reduction in plant biomass, compared with the inoculated control. However, a sole benzoic acid application in the absence of *R. solani* showed no influence on the total biomass of lettuce (Table 2). 

Two independent growth chamber experiments at two different temperature regimes with optimal (23–25 °C) and sub-optimal (20–22 °C) temperatures for *R. solani* infection [6] confirmed similar trends. Co-application of benzoic acid to the *R. solani*-inoculated plants resulted in increased shoot and root biomass and increased fine root lengths. Biomass production was generally lower at 20–22 °C as compared with higher temperature, 23–25 °C (Table 3). However, the protective effects of benzoic acid application against bottom rot disease caused by *R. solani* were greater under the less conductive temperature regime at 20–22 °C, particularly with respect to root growth promotion and fine root formation (Table 3).

### 2.4. Lettucenin A Distribution in Plant Tissues of Lettuce

**Figure 4:**
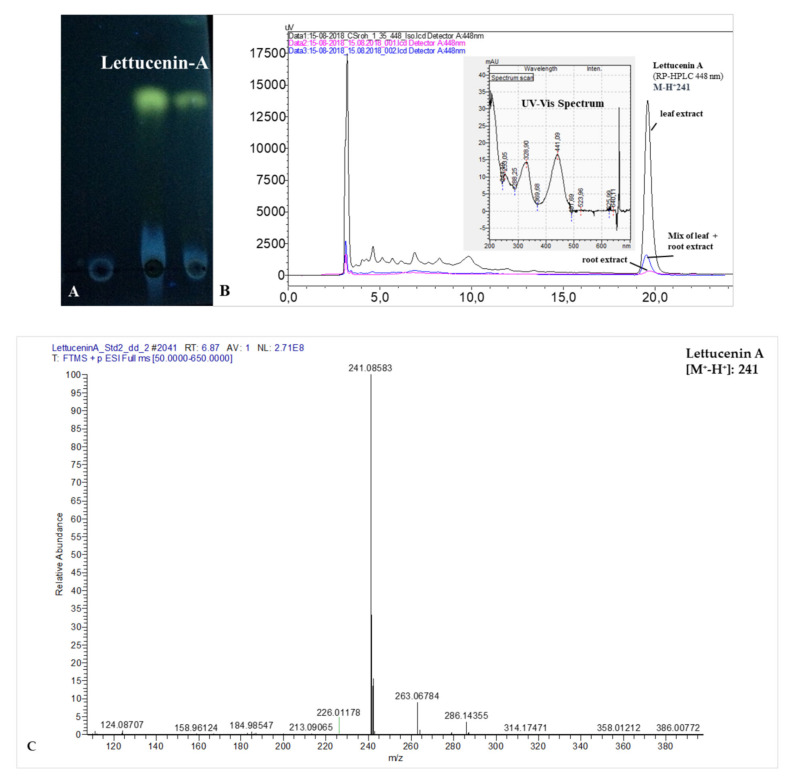
Detection of lettucenin A in leaf and root extracts of lettuce (cv. Tizian), using (**A**) thin-layer chromatography: yellow fluorescent lettucenin spots after elicitation of lettuce leaves with CuSO_4_ (left) and AgNO_3_ (right). (**B**) Identification of lettucenin A in leaf and root extracts of lettuce by comparison of retention times and spectral characteristics using RP-HPLC-UV/VIS and (**C**) by UHPLC orbitrap-MS.

Lettucenin A was identified by thin layer chromatography, RP-HPLC, spectral characteristics and UHPLC-MS in comparison with published data [31,32,33] as dominant lettucenin in the leaf tissues of lettuce and in lower concentrations in the root tissues after elicitation with CuSO_4_, AgNO_3_ and *R. solani* (Figure 4). 

In lettuce plants grown in peat culture substrate, a 46-fold increase in lettucenin A was detected in the leaf tissues treated locally with foliar sprays of CuSO_4_ as compared with the untreated control, whereas no increase was recorded in the root tissues and in untreated leaves (Table 4). In *R. solani*-inoculated plants, lettucenin A increased in the root tissues (2-fold) and in infected leaves (7-fold), but not in non-infected leaves. Co-application of the *R. solani*-inoculant with benzoic acid in real rhizosphere concentrations resulted in a 66% reduction in lettucenin A in the *R. solani*- infected leaf tissue compared with the treatment lacking benzoic acid supply (Table 4). 

### 2.5. Defense Response of Soil-Grown Lettuce against Olpidium sp. Depending on Fertilization History

A high incidence of the lettuce pathogen *Olpidium* sp. was detected by visual rating, particularly in lettuce roots grown in minirhizotrons in BIODYN2 soil with biodynamic fertilization history (Figure 3; Table 5), compared with CONMIN soil with long-term mineral fertilization (see Section 4.1.3). The investigated soils were collected from two long-term field experiments [20]. A high rhizosphere abundance of *Olpidium brassicae* (syn. *virulentus*) (76–90%) was confirmed earlier by amplicon sequencing in both soils [22]. However, typical fungal structures within the tips of fine roots associated with the loss of root hairs (Figure 3F) and intracellular formation of sporangia (Figure 3G) were detected, particularly in BIODYN2 soil. The preferential *Olpidium* infection recorded in the BIODYN2 soil was associated with a significant decline of total plant biomass (47%) as compared with the plants grown in CONMIN soil (Table 5). In accordance with the higher pathogen pressure in the roots of BIODYN2 plants compared with the plants grown in CONMIN soil, the rhizosphere concentration of benzoic acid, which was released as defense compound from root tips as major infection sites of *Olpidium*, increased by 203%. A similar trend was observed for lettucenin A, although with very low concentrations close to the detection limit. Benzoic acid was detectable exclusively in the root tissue of lettuce plants, grown in BIODYN2 soil. Lettucenin A accumulated both in root and leaf tissue, with higher levels in the roots and a trend for increased concentrations in plants grown in BIODYN2 soil as compared with the CONMIN soil (Table 5). 

## 3. Discussion

The concept of root exudates as a component of belowground plant defense responses against pathogens has a long history. However, exploring the profiles of secreted metabolites that exhibit a defensive function in the rhizosphere is technically much more challenging compared with the analysis of antibiotic compounds in aboveground plant parts [14]. In this study, we obtained more detailed insights into the role of root exudates in plant defense responses against pathogens in a well-characterized pathosystem with lettuce and *R. solani* [20,22,35] using a combined approach of model experiments in hydroponics, peat substrates and real soil culture. 

### 3.1. Benzoic Acid—A Root Exudate of Lettuce with Pathogen Defense Effect

Benzoic acid has been detected in the rhizosphere soil solution of soil grown lettuce plants in previous studies [17,20,22]. To address the question whether benzoic acid is a plant-released root exudate, we cultivated lettuce plants in a hydroponic culture system, avoiding soil contact and formation of a rhizosphere. Benzoic acid was detected by UHPLC-MS analyses after short-term immersion of the root systems into trap solutions (3 h to minimize microbial degradation, as a first indication that this compound was released from roots of lettuce plants (Figure 1; Table 1). Further, we detected benzoic acid also in the root tissue of the plants used for exudate collection. However, free benzoic acid was present only in trace concentrations in methanolic root extracts compared with significant amounts detected after alkaline hydrolysis of the extracts (Table 1). This finding suggests that benzoic acid in the root tissue is mainly present in form of benzoic acid esters to increase the water solubility for storage in the vacuole and reduce auto-toxic effects, as previously reported for benzoyl glucose in tobacco [36]. A release of free benzoic acid may then occur by enzymatic hydrolysis just prior or directly after release into the apoplast, similarly reported also for other secondary metabolites, such as flavonoid glycosides [37]. Since benzoic acid was detected in the esterified form in the root tissue of lettuce grown in hydroponics without any biotic or abiotic stress factor, this compound can be regarded as a phytoanticipin, which constitutively accumulates in plant tissue as a defense compound [38]. We speculate that the exudation in form of benzoic acid is triggered by a stimulation via fungal pathogens such as *R. solani* but also by inoculation with beneficial bacteria able to suppress diseases, as demonstrated by Windisch et al. [20]. This offers perspectives to manipulate root exudation of defense compounds by application of microbial inoculants for biocontrol approaches [21]. Nevertheless, under soil conditions, it cannot be ruled out that microbial benzoic acid production [26,27,28] could also have contributed to this effect, at least to some extent. However, this scenario still remains hypothetic, since in previous studies [20] it was not demonstrated that the concentrations measured for benzoic acid in the lettuce rhizosphere are sufficient to exert any inhibitory effects on growth and pathogenesis of the co-inoculated *R. solani* pathogen. 

To address this question, in our study, benzoic acid was applied in realistic rhizosphere concentrations, determined in earlier studies [22] to a PDA growth medium in a plating assay with *R. solani*. A significant reduction (30%) in fungal growth during 72 h (Figure 2) demonstrated an inhibitory effect of benzoic acid in a concentration range detectable in the rhizosphere of soil-grown lettuce. However, during longer incubation times the fungus was able to colonize the whole plate, pointing to a potential of benzoic acid degradation by fungal activity as demonstrated in previous studies [39]. 

To assess the effects of benzoic acid exudation on bottom rot disease caused by *R. solani*, benzoic acid was applied with the irrigation water to lettuce co-inoculated in the rhizosphere with the pathogen and grown in a peat-sand substrate (Figure 3, Table 2). First disease symptoms at the basal leaves were detectable already at two days after inoculation and clear bottom rot leaf lesions and root infection were recorded after five days (Figure 3A), which was associated with a significant plant biomass reduction of 41 g compared with the untreated control (Table 2). By contrast, the biomass of lettuce plants with *R. solani* inoculation and simultaneous application of benzoic acid was reduced only by 22 g, indicating a direct inhibitory effect against *R. solani*. Application of benzoic acid without *R. solani* inoculation had no effects on biomass production (Table 2) excluding any growth effects induced, e.g., by a potential bio-stimulant function of benzoic acid. 

In two independent experiments, particularly root growth of *R. solani*-inoculated plants was increased after benzoic acid application, demonstrating a pathogen-suppressive effect of benzoic acid in real rhizosphere concentrations (Table 3). The protective effects were more pronounced under sub-optimal temperature conditions for root infection with *R. solani* (20–22 °C), obviously weakening the pathogenic potential of the fungus. Similar protective effects of benzoic acid in root exudates have been reported against the bacterial tobacco pathogen *Ralstonia solanacearum* already at much lower soil concentrations of 4 µg kg^−1^ substrate [25].

### 3.2. Lettucenin A as Phytoalexin in Lettuce 

Strong local plant defense responses, which resulted in lettucenin accumulation, known as major phytoalexin in leaf tissues of lettuce, have been observed in earlier studies [31,33,40]. Accordingly, in our study we detected increased accumulation of lettucenin A in lettuce leaves as a response of plants to local chemical elicitation with CuSO_4_ and AgNO_3_ (Figure 4). In addition, we aimed to answer the question whether lettucenins, which have so far only been detected in leaves of lettuce, could also play a role in defense reactions in the root or even in root exudates of lettuce. Confirming the results of earlier studies [40], a local leaf elicitation with CuSO_4_ raised the lettucenin A accumulation exclusively in the treated leaves with a 46-fold increase as compared with the untreated control. However, no increase was recorded in the root tissues. By contrast, in the presence of *R. solani*, causing the so-called bottom rot disease in lettuce, affecting both roots and the lower leaves of lettuce plants (Figure 3A), increased lettucenin A levels were detected in the infected leaves and to a lower extent also in the root tissue (Table 4). To our knowledge, this is the first report of lettucenin A accumulating as a defense compound in roots of lettuce. Interestingly, application of benzoic acid as antimicrobial compound to *R. solani*-inoculated plants resulted in a 66% reduction in lettucenin A accumulation in the leaf tissue (Table 4). This may reflect the reduced disease severity observed in the respective plants due to the antagonistic effect of benzoic acid. Accordingly, in this case, the root and shoot biomass was significantly increased (Table 3). 

### 3.3. Chemical Defense Responses of Lettuce in Soil Culture 

We conducted an additional minirhizotron experiment under real soil conditions by growing lettuce plants in a silty loam from an experimental long-term field site, which was affected by the fungal lettuce pathogen *O. brassicae* [22]. Two strategies of long-term fertilization practices were compared, namely one with organic (BIODYN2), and the other with mineral (CONMIN) fertilization history. The experiment revealed severe growth depression of lettuce (Table 5) after root infection by the biotrophic pathogen *Olpidium brassicae* grown in the organically fertilized soil of BIODYN2 in comparison with CONMIN soil with mineral fertilization history. Microscopic root examination (Figure 3) and amplicon sequencing of fungal DNA in the respective rhizosphere soils [22] confirmed *Olpidium* infection of BIODYN2 plants. The intense *Olpidium* infection in the BIODYN2 treatment was associated with an increase in benzoic acid accumulation (203%) in the rhizosphere of 1 cm apical root zones of young roots (Table 4), known as major *Olpidium* infection sites (Figure 3E). The results suggest a defense response similar to the effects observed after *R. solani* inoculation [20,22]. However, Windisch et al. [22] did not observe a comparable increase in benzoic acid accumulation in the apical root zones of the lettuce plants grown in BIODYN2 soil. The discrepancy may have resulted from the longer culture period of nine weeks [22], compared with six weeks in this study. During longer lasting plant-pathogen interactions, the fungus was obviously able to overcome and suppress the defense response of lettuce with respect to benzoic acid release. Hence, plant growth was negatively affected (Table 5). Similar suppressive effects of pathogens, counteracting plant defense responses have been frequently reported, induced via production of effector proteins by various bacterial and fungal pathogens [41].The ability to suppress plant immunity via production of effector proteins is of particular importance for biotrophic pathogens, such as *O.brassicae*, depending on living host cells for successful colonization.

In contrast to benzoic acid, lettucenin A was detectable in trace amounts close to the detection limits in the rhizosphere soil solution of lettuce plants grown in CONMIN and BIODYN2 soils (Table 5). However, in accordance with the function as phytoalexin, lettucenin A accumulated preferentially in the root tissue of the plants, directly affected by the endophytic root pathogen *Olpidium*, whereas leaf concentrations only reached 30% of the lettucenin A levels detected in roots. The results suggest a relationship between disease severity and increased concentrations of lettucenin A in the rhizosphere soil solution (36%), root tissue (33%) and leaf tissues (61%) in the BIODYN2 soil compared with the less-affected plants grown in the CONMIN soil. However, the differences were not statistically significant (Table 5). The extremely low concentrations of lettucenin A close to the detection limits in the rhizosphere soil solution, showing similar treatment differences as compared with the root contents, suggest in contrast to benzoic acid, a rather passive (e.g., diffusion-mediated) release of lettucenin A than a controlled exudation in response to pathogen infection. 

## 4. Materials and Methods

### 4.1. Plant Cultivation in Hydroponics, Peat Culture Substrate and Soil Culture

To assess defense responses in lettuce (*Lactuca sativa* cv. Tizian, Syngenta, Bad Salzuflen, Germany) against pathogens, growth chamber experiments were performed, using hydroponics-, pot experiments with peat culture substrate and soil culture in minirhizotrons. 

#### 4.1.1. Pathogen-Suppressive Effect of Benzoic Acid

To achieve homogenous plant development, lettuce seedlings were pre-cultivated in growing trays until the five-leaf stage (BBCH 15; 7 weeks) in a pre-fertilized peat culture substrate (TKS1)–sand mixture (90/10: *w*/*w*). Thereafter, the seedlings were transferred to pots with 1 kg TKS1-sand mixture (Floragard, Oldenburg, Germany). The plants were watered to 70% of substrate water-holding capacity (WHC) with demineralized water throughout the culture period. Lettuce seedlings were cultivated in a growth chamber with a 16 h light period at 420 µmol m^−2^ s^−1^, 60% relative humidity, at 25 °C (experiment 1) and at 22 °C (experiment 2). 

For pathogen inoculation, a *R. solani* AG1-IB isolate 7/3 from the collection of the Leibnitz Institute of Vegetable and Ornamental Crops (Großbeeren, Germany) was pre-cultured on potato dextrose agar (PDA, Roth, Karlsruhe, Germany), supplemented with penicillin G (Roth, 100 mg L^−1^), streptomycin sulfate (Sigma-Aldrich, Taufkirchen, Germany; 50 mg L^−1^) and tetracyclin (Roth, 10 mg L^−1^) in petri dishes (9 cm diameter). The plates were incubated at 25 °C in darkness for 4 days (WTB Binder incubator, Tuttlingen, Germany). One day after transplanting (DAT), seven agar plugs (5 mm diameter) with fungal mycelium were excised from the plates, inserted into the peat culture substrate (1 cm deep) at a distance of 1–2 cm from the roots close to the rhizosphere of lettuce plants (BBCH 15) and covered with a thin layer of substrate to minimize evaporation. Non-infested agar pieces were used in the control treatments. Two independent pot experiments and a hydroponic experiment were conducted.

To investigate the pathogen-suppressive effect of benzoic acid at real rhizosphere concentrations, benzoic acid (pre-dissolved in an ethanolic stock solution at 2 mg L^−1^) was added with the demineralized irrigation water (1 mL stock solution L^−1^) to the lettuce plants inoculated with *R. solani* to reach a concentration of 110 µg kg^−1^ substrate. Control plants received the same amount of water with addition of pure ethanol. The respective concentration of benzoic acid was previously determined in the rhizosphere of lettuce grown on a loamy sand with suppressive potential against *R. solani* AG1-IB [22]. It corresponded to a benzoic acid concentration of approx. 500 µg L^−1^, which has been calculated for the rhizosphere soil solution in a distance of 1 mm from the root surface, equivalent to 110 µg kg^−1^ rhizosphere soil, assuming a volumetric soil water content of 20% and a root diameter of 1 mm [42,43]. Benzoic acid was applied three times after transplanting, to simulate the continuous release of benzoic acid from the plant roots in different stages of root development [20,22] and to account for microbial degradation during this time period [44], as suggested by the presence of bacterial rhizosphere responders of the genus *Sphingomonas* with the ability for degradation of aromatic hydrocarbons repeatedly identified in the rhzosphere of lettuce [21,22].

To induce the production of lettucenin phytoalexins, a chemical elicitation of lettuce plants by foliar sprays of three leaves with 5% (*w*/*v*) CuSO_4_ solution was conducted at one DAT in selected treatments, whereas the remaining leaves were covered with plastic foil during the spraying process to prevent contact with CuSO_4_. At 10 DAT, leaves were harvested and separated as “leaves with” and “without” symptoms of *R. solani* infection and CuSO_4_ elicitation, respectively. Fresh root biomass was determined and one-half was frozen in liquid nitrogen and kept in −80 °C for metabolite determinations. The other half was kept in 60% (*v*/*v*) ethanol for root morphology analysis.

#### 4.1.2. Benzoic Acid Released from Roots of Lettuce Cultivated in Hydroponics

After a pre-culture period in peat culture substrate and transplanting into pot culture as described in Section 4.1.1, the plants were carefully removed from the peat culture substrate at eight DAT by washing the root system with demineralized water. Thereafter, the plants (one plant per pot) were transferred to pots containing 2.8 L nutrient solution, continuously aerated with an aquarium pump (Eheim, Deizisau, Germany). Pathogen inoculation with *R. solani* was performed 6 days before transferring the plant into the hydroponic nutrient solution. The nutrient solution consisted of 1.0 µM H_3_BO_3_, 0.5 µM MnSO_4_, 0.5 µM ZnSO_4_, 0.2 µM CuSO_4_, 0.1 µM (NH_4_)_6_Mo_7_O_24_, 300 µM Fe-EDTA, 0.1 mM KH_2_PO_4_, 0.6 mM MgSO_4_, 2.5 mM Ca(NO_3_)_2_, 1.1 mM K_2_SO_4_. The nutrient solution was replaced every 48 h with half-strength mineral concentrations directly after transplanting to hydroponics and proceeded with full-strength nutrient solution during an eight days-culture period and a 22 °C/20 °C day/night temperature regime. Thereafter, root washings were collected by immersion of the root system into 300 mL aerated demineralized water for 3 h [45]. Finally, root and leaf biomass of the plants was recorded, and root washings and the root tissue were frozen at −80 °C for later analysis of benzoic acid.

#### 4.1.3. Lettuce Cultivation in Minirhizotrons with Soil Culture

For the minirhizotron experiment, lettuce seeds were sown in seedling trays filled with soils of different fertilization history and pre-cultivated until the five-leaf stage (BBCH 15). Thereafter, lettuce seedlings were transplanted to minirhizotrons filled with 0.6 kg of soil-sand mixture 70/30 (*w*/*w*) equipped with removable root observation windows to enable micro-sampling of rhizosphere soil solution by application of sorption filters onto the surface of roots growing along the observation windows as described by Windisch et al. [20,22]. The soils originated from a long-term field experiment DOK-LTE conducted by the Research Institute of Organic Agriculture (FIBL; Therwil; Switzerland) since 1978 on a Haplic Luvisol (silty loam), and compares long-term bio-dynamic compost and manure fertilization (BIODYN2) with mineral NPK (CONMIN) fertilization. Detailed soil characteristics, management practices and physicochemical parameters of the experimental soils are described in Windisch et al. [22]. Lettuce plants were cultivated under growth chamber conditions according to Windisch et al. [22] with four replicates per treatment. Regular watering of the plants was supplied to reach 60% of substrate WHC by addition of demineralized water. Microscopic evaluation of root pathogens was performed at 21 DAT with a subset of lettuce plants as described by Windisch et al. [22]. Micro-sampling of rhizosphere soil solution with sorption filters [17] was conducted at 31 DAT during 4 hours in 1 cm subapical root zones (1–2 cm behind the root tip) of young, growing roots considered as the root zones with the highest release rates of low molecular-weight root exudates [42]. For each minirhizotron, the filters of five sampling points were pooled and stored frozen at −20 °C. Thereafter, final harvest was performed with determination of shoot (total fresh shoot biomass) and root biomass. Leaves and roots were frozen in liquid nitrogen and stored at −80 °C for further analysis.

### 4.2. Plating Assay for the Effect of Benzoic Acid on R. Solani

To assess the pathogen suppressive potential of benzoic acid, different dosages of benzoic acid were incorporated after sterile filtration (0.2 µm) into PDA medium at 40 °C to reach final concentrations of 0 mg L^−1^, 0.05 mg L^−1^ and 0.5 mg L^−1^, respectively. An agar plug of an actively growing *R. solani* culture without benzoic acid was transferred onto the middle of the PDA plate using a sterile inoculation loop to assess the ability of the fungus to grow into a medium with different benzoic acid concentrations. Fungal hyphae, trying to spread into the lettuce rhizosphere with benzoic acid accumulation would face a very similar situation. The mycelial growth of the fungus on the agar plates was quantified by determining the colony diameters during a cultivation period of three days at 25 °C with three replicates per treatment. The mycelial growth inhibition percentage was calculated. 

### 4.3. Benzoic Acid in Root Washings, Rhizosphere Soil Solution and Plant Tissues

Benzoic acid in root washings of lettuce plants grown in hydroponics (Section 4.1.2) was pre-purified via solid-phase extraction with Sep-Pac C18 cartridges (Waters Corporation, Milford, MA, USA). Conditioning of the Sep-Pac C18 cartridges was conducted with 5 mL of MeOH (100%). A 20 mL aliquot of root washing solutions was passed through the cartridge at a flow rate of 3 mL min^−1^. Subsequent elution of hydrophobic compounds bound to the cartridge was performed with 5 mL of methanol: ethyl acetate (1:1; *v*:*v*) at a flow rate of 1 mL min^−1^. The eluted fraction was evaporated to dryness at 30 °C with Speed-vac concentrator (Savant, Farmington, CT, USA) and the pellet was re-dissolved in 200 µL of acetonitrile: H_2_O (1:4; *v*:*v*) and subjected to UHPLC-MS analysis using a Velos LTQSystem (Thermo Fisher Scientific, Waltham, MA, USA) equipped with an YMC Triart C18 column, 3 µm particle size, 100 mm × 3 mm (YMC Europe, Dinslaken, Germany) as described by Windisch et al. [22]. Rhizosphere soil solution, collected with sorption filters (Section 4.1.3) was extracted with 0.6 mL of acetonitrile: H_2_O (1:1; *v*:*v*) and aliquots of 50 µL obtained were analyzed by UHPLC-MS [22].

For determination of benzoic acid in plant tissues, 2 g of frozen leaf and root tissue were homogenized with liquid nitrogen and extracted in 4 mL methanol: H_2_O (4:1; *v*:*v*) using mortar and pestle. After centrifugation for 5 min at 12,000 rpm (Microliter centrifuge Micro 24–48 R, Hettich, Tuttlingen, Germany), the supernatant was subjected to membrane filtration (PTFE 0.2 µm pore size, Roth, Karlsruhe, Germany). Aliquots of the tissue extracts (100 µL) were mixed with 300 µL demineralized of H_2_O, followed by UHPLC-MS analysis [22]. To release conjugated benzoic acid, alkaline hydrolysis of the root extracts was performed with 2N NaOH at pH 8.0 for 30 min at room temperature according to Chong et al. [36]. The hydrolysate was subjected twice to liquid–liquid extraction with 2 mL ethyl acetate, and the combined upper phases were evaporated to dryness by nitrogen vaporization (Multivap 11880, Organomation, Berlin, Germany), followed by dissolving in 200 µL of acetonitrile: H_2_O (1:4; *v*:*v* ) for UHPLC-MS analysis [22].

### 4.4. Determination of Lettucenin A in Plant Tissues and Root Exudates

Frozen leaf and root material (1 g) was homogenized in liquid nitrogen and extracted with 2 mL of MeOH: H_2_O (4:1; *v*:*v*) using mortar and pestle, followed by centrifugation for 5 min^−1^ and 12,000 rpm. 1.5 mL aliquots of the supernatants were membrane filtered (PTFE 0.2 µm pore size, Roth, Karlsruhe, Germany) for further analyses with RP-HPLC (Shimdazu High performance LC20 system, Kyoto, Japan). The identification and quantitative determination of lettucenin A was conducted, using a reversed phase C-18 column (GROM-SIL 120 ODS, 5 µm particle size, 290 mm × 4.6 mm equipped with a 20 mm × 4.6 mm guard column with the same stationary phase (Dr. Maisch HPLC GmbH, Ammerbuch, Germany). The UV detection was conducted at 448 nm and isocratic elution with 50% (*v*:*v*) methanol: water at a flow rate of 0.7 mL min^−1^, column temperature of 35 °C and an injection volume of 20 µL. The identification and quantitative analysis were performed by comparison of spectral characteristics and retention time with an external lettucinin A standard. 

To obtain a pure lettucenin A standard after methanolic extraction of 2 g frozen leaf material (as described above), a solvent extraction of the methanolic extract was performed three times with 2 mL ethyl acetate each. The combined upper phases were evaporated to dryness at 30 °C using a Speed-vac concentrator (Savant, Farmington, CT, USA), followed by re-dissolving the residue in 150 µL of methanol and further purification by thin-layer chromatography (TLC) according to Yean et al. [32] and Talubnak et al. [40]. An aliquot of 30 µL methanol extract was applied onto a TLC plate (ALUGRAM SIL G/UV_254_, 5 × 10 cm, Macherey-Nagel, Düren, Germany) and the separation was performed with a solvent system of hexane: ethyl acetate (1:1; *v*:*v*). Lettucenin A was detected as bright yellow florescent band (Rf 0.47), when examined under UV light at 365 nm wavelength. The yellow band was scratched from the TLC plate and re-extracted with 0.5 mL methanol. After 5 min centrifugation at 14,000 rpm to remove the silica particles, the supernatant was checked for purity by RP-HPLC as described above (Figure 4A). Identity was confirmed by comparison of UV-Vis absorption spectra with published data [31,33] and by mass spectrometry analysis using a Agilent 1290 U-HPLC system (Agilent Technoligies Inc., Palo Alto, USA) coupled to a QExactive Plus Orbitrap quadropoly-mass spectrometer (Thermo Fisher Scientific, Dreieich, Gemany). The separation was performed on an Acquity UPLC CSH C18 column (Waters Acquity, Milford, MA, Unites States, 1.7 µm, 2.1 × 150 mm). The elution was performed with (A) formic acid aqueous solution and (B) methanol using a gradient elution of 10% B at 0, 10−90% B at 0−10 min and 90% B at 10−15 min. Full scan mass spectra (ESI, mass range *m*/*z* 50–650) of HPLC eluates were recorded during chromatographic separation in the positive ionization mode. 

The lettucenin A concentration in the purified standard solution was calculated via Lambert-Beer’s law based on spectrophotometric determination (Hitachi U-3300 spectrophotometer, Hitachi Ltd., Corporation, Tokyo, Japan) of the absorption at 446 nm and the published absorption coefficient Ɛλ = 32,000 [31] and the respective standard was used for quantitative determinations. 

Lettucenin A in the rhizosphere soil solutions, collected in the minirhizotron experiment (Section 4.1.3) was determined together with benzoic acid by UHPLC-MS as described in Section 4.3.

### 4.5. Statistical Analyses

The experiments in pots, minirhizotrons and hydroponics were carried out in a completely randomized block design with one lettuce plant for each treatment in four and five replicates, respectively. Differences between treatment groups were analyzed using ANOVA followed by Tukey’s test and Tukey’s HSD pairwise testing (*p* ≤ 0.05 significance level). Differences in lettucenin A concentration between leaf and root tissues were analyzed by *t*-test. Calculations were done in R studio (Ri386 3.4.0). 

## 5. Conclusions

The presented data support the hypothesis, that benzoic acid is released from lettuce roots as a defense compound in response to fungal pathogen attack, which originates from esterified precursors pre-accumulating in the root tissue. At least in the investigated lettuce-*R. solani* plant–pathogensystem, the accumulated rhizosphere concentration of benzoic acid was sufficient to inhibit mycelial growth of the fungal pathogen and reduced the disease severity of infected plants. However, in the soil culture experiments, a certain contribution of microbial benzoic acid production cannot be completely excluded. In contrast to benzoic acid, lettucenin A accumulates preferentially as phytoalexin in infected tissues. However, it was not detectable in significant amounts as root exudate. These findings suggest that the release of benzoic acid represents a first defense line upon pathogen attack in the rhizosphere, followed by local accumulation of the lettucenin A phytoalexin within the affected tissue as a second line of defense. For the first time it was demonstrated that lettucenin A is not only produced as phytoalexin upon pathogen infection in the leaf tissue of lettuce as reported in earlier studies but similarly accumulates in affected roots. However, the pathogen suppressive potential of the lettucenin A accumulation against the investigated pathogens still remains to be investigated.

The possibility to manipulate the defense responses by chemical elicitation or inoculation with beneficial microorganisms may offer perspectives for biocontrol strategies. However, the observed variability in responses with respect to the type of pathogens, soil type [7,20,21] and temperature effects (Table 3) indicates that detailed knowledge on successful application conditions is indispensable.

## Figures and Tables

**Table 1:** Detection of benzoic acid in root washings of lettuce (cv. Tizian) (**A**) and in root tissue before and after alkaline hydrolysis of methanolic plant extracts (**B**). Means ± SE of four replicates per treatment. Different lowercase letters indicate significant differences between treatments by one-way ANOVA, Tukey’s test (*p* ≤ 0.05).

Benzoic Acid [ng g^−1^ Root FW]		
A. Root Exudate	B. Root Tissue	
	Methanolic extract before alkaline hydrolysis	Methanolic extract after alkaline hydrolysis
0.04 ± 0.03 a	0.03 ± 0.01 a	0.20 ± 0.02 b

**Table 2:** Decline in total fresh weight (FW) of lettuce (cv. Tizian) grown in peat culture substrate at 25 °C for 10 days with (i) application of benzoic acid (3 × 110 µg kg^−1^ substrate throughout growing period via irrigation), (ii) with *R. solani* AG1-IB inoculation and (iii) combined application of benzoic acid and *R. solani*, compared with an untreated control. * = significant difference compared with the untreated control; ** = significant difference compared with the untreated control and the *R. solani* treatment; one-way ANOVA, Tukey’s test (*p* ≤ 0.05).

Parameter	(i) Benzoic Acid Application [3 × 110 µg kg^−1^ Substrate]	(ii) *R. solani* Inoculation	(iii) *R. solani* + Benzoic Acid Application [3 × 110 µg kg^−1^ Substrate]
Decline in plant FW (compared with an untreated control) [g plant^−1^]	0.29	44.22 (*)	21.6 (**)

**Table 3:** Fresh weight (FW) of shoot and roots, root and fine root length of lettuce (cv. Tizian) grown for 10 days in peat culture substrate in two independent growth chamber experiments at different temperature regimes (23–25 °C and 20–22 °C). Plants were inoculated with *R.* *solani* AG1-IB with and without application of benzoic acid (3 × 110 µg kg^−1^ substrate throughout the growing period via irrigation). In each row, different lowercase letters indicate significant differences according to one-way ANOVA, Tukey’s test (*p* ≤ 0.05).

Incubation Temperature	23–25 °C		20–22 °C	
Treatments	*R. solani*	*R. solani* + Benzoic Acid	*R. solani*	*R. solani* + Benzoic Acid
Shoot FW [g]	113.73 a	131.62 b (+16%)	83.39 a	94.36 a (+13%)
Root FW [g]	9.82 a	11.58 b (+18%)	5.43 a	8.23 b (+52%)
Root length [cm]	1429.2 a	1510.7 a (+ 6%)	1196.7 a	2631.5 b (+120%)
Fine root length (Ø 0–0.4 mm) [cm]	1022.7 a	1182.1 a (+ 16%)	863.0 a	2040.3 b (+136%)

**Table 4:** Lettucenin A content in leaf and root tissue of lettuce (cv. Tizian), grown for 10 days in peat culture substrate after treatment with biotic (*R. solani* AG1-IB inoculation) and abiotic (CuSO_4,_ 5% *w*/*v* foliar sprays) elicitors and benzoic acid application (3 × 110 µg kg^−1^ substrate throughout the growing period via irrigation) and benzoic acid application with *R. solani* inoculated plants. Means ± SE of four replicates per treatment. Significant differences (*t*-test, *p* ≤ 0.05) compared with untreated control plants are indicated by *; na = not applicable.

	Analysed Plant Tissue			
Treatments	Leaves without Symptoms	Leaves with Symptoms of *R. solani*	CuSO_4_ Treated Leaves	
	Leaf Content [µg g^−1^ FW]			Root Content [µg g^−1^ FW]
Untreated control	0.17 ± 0.01	na	na	0.18 ± 0.01
CuSO_4_ foliar application	0.17 ± 0.06	na	7.96 ± 0.79 *	0.20 ± 0.01
*R. solani* inoculation	0.00	1.20 ± 0.68	na	0.42 ± 0.04 *
Benzoic acid application	0.21 ± 0.01 *	na	na	0.00
*R. solani +* Benzoic acid	0.12 ± 0.07	0.41 ± 0.13 *	na	0..65 ± 0.14 *

**Table 5:** Plant biomass, visual rating of *Olpidium* disease severity, lettucenin A and benzoic acid concentrations in root exudates, root and leaf tissues of lettuce (cv. Tizian) grown in minirhizotrons in soils collected from fields with long-term biodynamic (BIODYN2) vs. mineral (CONMIN) fertilization history. Means of five replicates per treatment. Different lowercase letters indicate significant differences between biodynamic vs. mineral fertilization by one-way ANOVA, Tukey’s HSD pairwise test, (*p* ≤ 0.05). Disease severity’ assessed by +++ high and + low expression of disease symptoms on roots.

Treatments	*Olpidium*- Disease Severity	Plant Biomass	Root Exudates		Root Tissue		Leaf Tissue	
	(Visual Rating)	[g Plant^−1^]	Lettucenin A [ng cm^−1^ Root]	Benzoic Acid [ng cm^−1^ Root]	Lettucenin A [µg g^−1^ FW]	Benzoic Acid [µg g^−1^ FW]	Lettucenin A [µg g^−1^ FW]	Benzoic Acid [µg g^−1^ FW]
BIODYN2 CONMIN	+++	1.41 b	0.34 a	4.06 b	1.77 a	0.11 a	0.49 a	0.0 a
	+	2.64 a	0.25 a	1.34 a	1.33 a	0.0 b	0.30 a	0.0 a
